# P43 Chronic TB cases constitute a high-risk population for the development and transmission of XDR TB

**DOI:** 10.1093/jacamr/dlaf230.050

**Published:** 2025-12-04

**Authors:** Platon Eliseev, Evgeny Kuznetsov, Ekaterina Gosteva, Natalya Gorshkova, Anastasia Samoilova, Vadim Testov, Irina Vasilyeva

**Affiliations:** The National Medical Research Center of Phthisiopulmonology and Infectious Diseases (NMRC PhPI) under the Ministry of Health of the Russian Federation, Moscow, Russia; The National Medical Research Center of Phthisiopulmonology and Infectious Diseases (NMRC PhPI) under the Ministry of Health of the Russian Federation, Moscow, Russia; The National Medical Research Center of Phthisiopulmonology and Infectious Diseases (NMRC PhPI) under the Ministry of Health of the Russian Federation, Moscow, Russia; The National Medical Research Center of Phthisiopulmonology and Infectious Diseases (NMRC PhPI) under the Ministry of Health of the Russian Federation, Moscow, Russia; The National Medical Research Center of Phthisiopulmonology and Infectious Diseases (NMRC PhPI) under the Ministry of Health of the Russian Federation, Moscow, Russia; The National Medical Research Center of Phthisiopulmonology and Infectious Diseases (NMRC PhPI) under the Ministry of Health of the Russian Federation, Moscow, Russia; The National Medical Research Center of Phthisiopulmonology and Infectious Diseases (NMRC PhPI) under the Ministry of Health of the Russian Federation, Moscow, Russia

## Abstract

**Background:**

The primary challenge for global and national TB control programmes is TB resistant to rifampicin and isoniazid (MDR-TB), including cases with additional resistance to fluoroquinolones (pre-XDR-TB). Treatment regimens for both MDR-TB and pre-XDR-TB include chemotherapy protocols containing bedaquiline and linezolid. A key aspect of the effective use of these drugs in TB control programmes is the low level of drug resistance to them. Prescribing effective treatment regimens is essential for reducing the burden of drug-resistant TB and preventing the spread of XDR-TB, particularly in countries with a high burden of MDR-TB. Currently, phenotypic methods are the main approach for drug susceptibility testing (DST) of bedaquiline and linezolid.

**Objectives:**

To determine the level of drug resistance to bedaquiline and linezolid (XDR) among various categories of pre-XDR-TB patients.

**Materials and methods:**

Data on the number of TB cases and DST results were obtained from the Federal TB Register in 2024. We determined the coverage and results of drug susceptibility testing for bedaquiline and linezolid among new cases, relapses and chronic cases of TB with pre-XDR.

**Results:**

In 2024, 36 104 new TB cases were registered in the Russian Federation. Bacteriological confirmation was obtained in 53.0% of cases (19 124/36 104). Among confirmed cases, resistance to fluoroquinolones (pre-XDR) was detected in 8.3% (1589/19 124) of patients. Among patients with pre-XDR-TB, testing for bedaquiline resistance was performed in 80.8% of cases (1284/1589), with resistance detected in 6.5% (84/1284). Testing for linezolid resistance was performed for 81.2% (1290/1589) of patients, with resistance found in 3.3% (42/1290) of cases. Among patients with TB relapses (*n*=8711), bacteriological confirmation was obtained in 56.9% of cases (4953/8711). The frequency of pre-XDR in this group was 18.4% (911/4953). The coverage of testing for bedaquiline and linezolid resistance was 82.9% (755/911) and 83.9% (764/911), respectively. The resistance rate to bedaquiline was 7.3% (55/755) and to linezolid - 6.0% (46/764). In the group of chronic TB patients (*n*=11 217), bacteriological confirmation was obtained in 55.8% of patients (6262/11 217). The proportion of pre-XDR was highest (*P<*0.05) in this cohort - 28.1% (1763/6262). The coverage of testing for bedaquiline and linezolid resistance was 72.1% (1272/1763) and 73.5% (1295/1763), respectively. The frequency of resistance to bedaquiline reached 12.1% (154/1272) and to linezolid - 7.6% (99/1295).

**Conclusions:**

This analysis demonstrates significant heterogeneity in the drug resistance structure of *Mycobacterium tuberculosis* depending on patient categories. The prevalence of pre-XDR and XDR-TB was statistically significantly lower among newly diagnosed cases compared to patients with TB retreatment and especially chronic TB patients. Determining drug resistance to bedaquiline and linezolid is essential for various cases of chronic TB to prescribe adequate personalized treatment regimens.

